# Source-sink modifications affect leaf senescence and grain mass in wheat as revealed by proteomic analysis

**DOI:** 10.1186/s12870-020-02447-8

**Published:** 2020-06-05

**Authors:** Xuemei Lv, Yan Zhang, Yunxiu Zhang, Shoujin Fan, Lingan Kong

**Affiliations:** 1 Crop Research Institute, Shandong Academy of Agricultural Sciences, Jinan, 250100 China; 2 College of Life Science, Shandong Normal University, Jinan, 250014 China

**Keywords:** Defoliation, Half-degraining, iTRAQ, Leaf senescence, Photosynthesis, *Triticum aestivum*

## Abstract

**Background:**

The grain yield of cereals is determined by the synergistic interaction between source activity and sink capacity. However, source-sink interactions are far from being fully understood. Therefore, a field experiment was performed in wheat to investigate the responses of flag leaves and grains to sink/source manipulations.

**Results:**

Half-degraining delayed but partial defoliation enhanced leaf senescence. Sink/source manipulations influenced the content of reactive oxygen species in the flag leaf and the concentration of phytohormones, including cytokinins, indoleacetic 3-acid and jasmonic acid, in the flag leaves (LDef) and grains (GDef) in defoliated plants and flag leaves (LDG) and grain (GDG) in de-grained plants. Isobaric tag for relative and absolute quantitation (iTRAQ)-based quantitative proteomic analysis indicated that at 16 days after manipulation, a total of 97 and 59 differentially expressed proteins (DEPs) from various functional categories were observed in the LDG and LDef groups, respectively, compared with the control, and 115 and 121 DEPs were observed in the GDG and GDef groups, respectively. The gene ontology annotation terms of the DEPs mainly included carbon fixation, hydrogen peroxide catabolic process, chloroplast and cytoplasm, oxidoreductase activity and glutamate synthase activity in the flag leaves of manipulated plants and organonitrogen compound metabolic process, cytoplasm, vacuolar membrane, CoA carboxylase activity, starch synthase activity and nutrient reservoir activity in the grains of manipulated plants. KEGG pathway enrichment analysis revealed that photosynthesis, carbon, nitrogen and pyruvate metabolism and glycolysis/gluconeogenesis were the processes most affected by sink/source manipulations. Sink/source manipulations affected the activities of amylase and proteinases and, ultimately, changed the mass per grain.

**Conclusions:**

Manipulations to change the sink/source ratio affect hormone levels; hydrolytic enzyme activities; metabolism of carbon, nitrogen and other main compounds; stress resistance; and leaf senescence and thus influence grain mass.

## Background

The grain yield of cereals is determined by the synergistic interaction between source activity and sink capacity. The source restores and supplies reserves to the sink (developing grains). Sink strength is determined by the number and potential size of grains per stem, depending on the capacity of plants to actively obtain photosynthetic assimilates and reserves in their vegetative organs and accumulate these compounds.

Wheat (*Triticum aestivum* L.) productivity is generally considered to be sink-limited under favorable conditions, with grain development regulated by the assimilating capacity but hardly limited by the source [1], because the source generally has the capacity to provide adequate assimilates to developing grains. An increase in the source/sink ratio does not affect the grain mass [2, 3]. Defoliation in an old germplasm of bread wheat does not cause a source limitation to grain filling [4]. However, inconsistent conclusions have also been observed. Kruk et al. [4] and Beed et al. (2007) [5] reported that grain yields are colimited by both the source and the sink in wheat. Experiments that manipulated assimilate availability during grain filling showed that wheat yields are mainly limited by the sink size [6]. Slafer and Savin reported that the grain yield of wheat is either sink-limited or colimited by both the source and sink but is never source-limited [7]. However, it is difficult to devise direct approaches to determine the relative importance of the source capacity and sink strength in contributing to the grain yield in wheat [8]. Therefore, a better understanding of source-sink interactions would help to propose strategies to improve yield potential using genetic approaches.

A number of phytohormones play vital roles in regulating leaf senescence and grain filling in crops as signaling molecules. Different roles of phytohormones in senescence have been observed: ABA induces senescence, while cytokinins (CTKs) and auxin inhibit senescence [9–11]. The cross-talk among various hormones has been outlined in previous studies. Hormones can be directly involved in the regulation of senescence or can function antagonistically. In addition, interactions of phytohormones with other factors, such as those involved in nitrogen status and sugar signaling, play critical roles in regulating source and sink communication [12, 13]. Therefore, the regulation of senescence by hormones is a complicated process that requires deeper investigation.

Isobaric tag for relative and absolute quantitation (iTRAQ) is an isobaric labeling method which has been popularly used to identify proteins coming from different sources, providing more reliable quantitative changes than traditional protein analysis [14]. Based on these identified proteins, metabolic pathways can be constructed and protein-protein interaction (PPI) analyses can be performed [15]. Proteomics studies in wheat using iTRAQ have primarily been performed to investigate protein responses to stresses such as drought, reactive oxygen species (ROS) stress and nutrient deficiency to assess the effects of environmental factors on profile characteristics and metabolic pathways [15, 16]. To date, however, quantitative proteomics studies based on iTRAQ analyses of wheat senescence and grain development when subjected to sink-source manipulations have not been reported.

Modifications of the source-sink relationship by changing the level of competition among developing grains and/or the assimilate availability have previously been used as manipulations after grain setting to understand the effects of the source/sink balance on grain development, aiming at suggesting strategies to increase the grain yield of wheat [7, 17, 18] and barley (*Hordeum vulgare*) [19]. Unfortunately, although much effort has been made, we are still far from fully understanding source-sink interactions [9]. How source-sink interactions can be regulated by cultivation methods and genetic manipulations remains unclear.

The present study was designed to examine the impact of manipulating the source/sink ratio on physiological modifications and protein expression in flag leaves and grains, aiming to determine how leaf senescence and grain mass are regulated by changing the availability of potential assimilates per grain. These results might promote a better understanding of the roles of the source-sink relationship in grain development and in finding effective avenues, such as genetic modifications during breeding, to further increase the cereal grain yield.

## Results

### Values of the normalized difference vegetation index (NDVI), photochemical reflectance index (PRI) and soil and plant analyzer development (SPAD)

The changes of the NDVI, PRI and SPAD values of flag leaves exhibited similar trends (Table 1). These parameters gradually decreased with the progress of grain filling. The PRI values showed greater sensitivity to sink-source modifications than the SPAD and NDVI values. Significant differences in PRI values were observed among the three treatments and growth stages. However, differences in NDVI values were only observed between half-degraining and defoliation at 16 and 24 days after manipulation (DAM). At 16 DAM, significant differences in SPAD values were only observed between half-degraining and defoliation. At 24 DAM, the highest level was observed in the half-degrained flag leaf (LDG) group, followed by a significant decrease in the flag leaves in control plants (LC) group and a further decrease in the defoliated flag leaf (LDef) group (Table 1).

**Table 1:** Effects of sink-source manipulations on the PRI, NDVI and SPAD values of flag leaves

DAM		PRI	NDVI	SPAD
8	Control	0.030b	7.98ab	58.77ab
	Half-degraining	0.032a	8.14a	59.83a
	Defoliation	0.028b	7.89ab	58.07ab
16	Control	0.026 cd	7.47 cd	57.27bc
	Half-degraining	0.028bc	7.74bc	58.03ab
	Defoliation	0.024e	7.26d	56.03c
24	Control	0.021f	6.21f	38.43e
	Half-degraining	0.025de	6.57e	40.73d
	Defoliation	0.018 g	6.14f	33.17f

PRI, Photochemical Reflectance Index; NDVI, Normalized Difference Vegetation Index; SPAD, Soil and Plant Analyzer Development. Data were obtained from three replicates. Means followed by the same letters within a column are not significantly different according to Duncan’s multiple range test (*p* < 0.05)

### Chlorophyll fluorescence

**Fig. 1:**
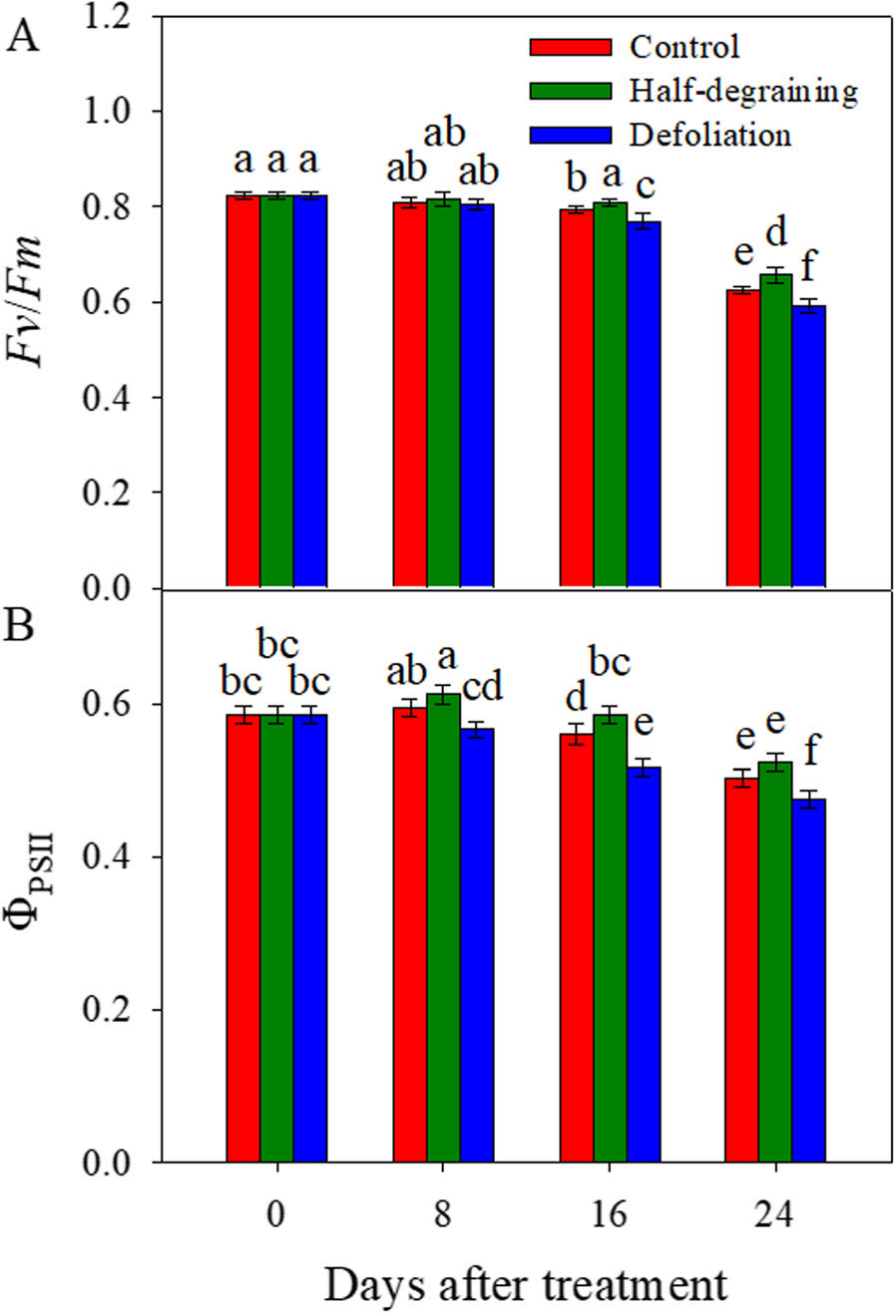
Effects of sink/source manipulation on the fluorescence parameters of flag leaves in wheat. *Fv/Fm*: maximal efficiency of the PSII photochemistry; Φ_PSII_: actual PSII efficiency. Each value is the mean ± standard deviation (SD) from at least six leaves. Columns labeled with different letters significantly differ at *p <* 0.05 according to Duncan’s multiple range test

Values of the maximum PSII quantum yield (*Fv*/*Fm*) and the effective PSII quantum yield (Φ_PSII_) of flag leaves were measured from 9:00–11:00 a.m. These values after the half-degraining treatment were higher than those of the intact control at 16 and 24 DAM. Defoliation significantly decreased the *Fv/Fm* value at 16 and 24 DAM and Φ_PSII_ value at 8, 16 and 24 DAM (Fig. 1).

### Chloroplast structure

**Fig. 2:**
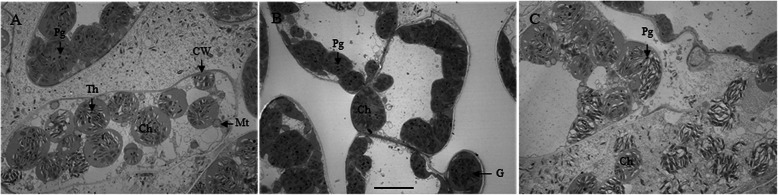
Transmission electron micrographs showing the ultrastructure of the chloroplast at 16 DAM in flag leaves of the control (**a**), half-degraining (**b**) and defoliation (**c**) treatment groups. The images are representative of five biological replicates. Bars: 5 μm; Ch, chloroplast; CW, cell wall; G, granum; Mt, mitochondrion; Pg, plastoglobuli; Th, thylakoid

An obvious difference in the cell ultrastructure of leaf tissues was observed under a transmission electron microscope at 16 DAM (Fig. 2). The chloroplasts in leaf tissues were spherical after all three treatments. In LDG cells (Fig. 2a), the chloroplasts contained a larger number of thylakoids and the matrix of the chloroplasts was still dense compared with those of the control (Fig. 2b) and defoliation treatment groups (Fig. 2c). In LDef cells, numerous small vesicles or membrane-like fragments were observed in the cytoplasm (Fig. 2c). The membranes constituting the thylakoids were less distinct and seriously disrupted in LDef cells compared to those in control cells. Moreover, the structure of the thylakoids in LDef cells was characterized by the loss of the parallel arrangement of the grana lamellae in some chloroplasts, and some of the thylakoids in these cells became swollen. In LDef cells, the number of chloroplasts was 9.80 ± 0.80 per cell section, significantly lower than 12.60 ± 1.39 and 13.67 ± 1.40 per cell section in LC and LDG cells (Fig. 2c), respectively.

### Antioxidant enzyme activity of flag leaf

**Fig. 3:**
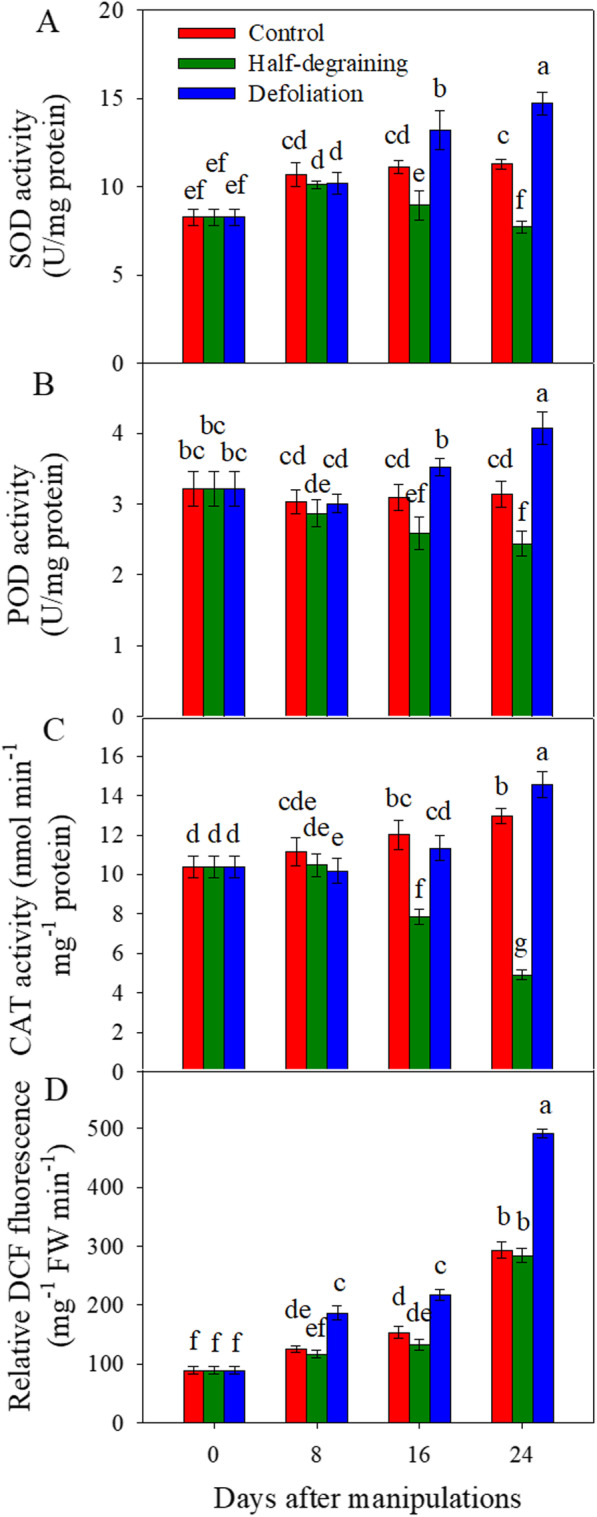
Effects of sink-source manipulations on antioxidant enzyme activities and the relative DCFH-DA fluorescence intensity of wheat flag leaves. The results are the mean ± SD

At 8 DAM, no significant difference in superoxide dismutase (SOD) activity was observed among the LDG, LDef and LC groups. At 16 and 24 DAM, half-degraining significantly decreased SOD activity compared with that of the control (*p <* 0.05). However, defoliation increased SOD activity at 16 (*p* > 0.05) and 24 (*p <* 0.05) DAM (Fig. 3a). Peroxidase (POD) activity exhibited an almost identical changing trend as SOD activity during the period from 8 to 24 DAM (Fig. 3b). At 8 and 24 DAM, half-degraining significantly decreased catalase (CAT) activity with respect to that of the control (*p <* 0.05). The defoliation treatment decreased CAT activity at 8 DAM (*p <* 0.05) but caused sharp increases in activity thereafter and thus induced a significant enhancement of CAT activity 24 DAM (*p <* 0.05) (Fig. 3c). Despite the higher activities of these enzymes, especially at 24 DAM, the ROS content was still higher in the flag leaves of defoliated plants compared to that in the flag leaves of the control. However, no difference in ROS content was observed in flag leaves between half-degraining and intact plants (Fig. 3d).

### Effects of sink-source manipulations on endogenous hormones in flag leaves and grains

The leaf/grain ratio of endogenous hormone concentration was used to evaluate the source strength and sink capacity balance. The total concentration of zeatin + zeatin riboside + kinetin was used to evaluate the changes in CTKs after manipulations. Compared with the control plants, the half-degraining treatments significantly increased and defoliation decreased the leaf/grain ratio of the CTK concentration at the three time points (*p <* 0.05) (Table 2). At 8 and 16 DAM, no significant difference in the leaf/grain ratio of indoleacetic 3-acid (IAA) concentration was observed between the degraining treatment and control. For defoliation, this treatment significantly decreased the leaf/grain ratio of IAA (*p <* 0.05) (Table 2). Half-degraining did not affect the leaf/grain ratio of jasmonic acid (JA) concentration, whereas defoliation significantly decreased this ratio throughout the experimental period (*p <* 0.05) (Table 2).

**Table 2:** Effects of sink and source manipulations on the phytohormone concentration leaf/grain ratio in wheat. Data were obtained from three replicates

DAM	Treatment	CTKs	IAA	JA
8	Control	0.75bc	0.76a	2.00a
	Half-degraining	0.99a	0.76a	1.65b
	Defoliation	0.57d	0.61 cd	0.84d
16	Control	0.70c	0.75a	1.65b
	Half-degraining	0.78b	0.72ab	1.58b
	Defoliation	0.52d	0.38e	0.65d
24	Control	0.82b	0.66bc	1.15c
	Half-degraining	0.95a	0.56d	1.07c
	Defoliation	0.70c	0.30f	0.71de

DAM: Days after manipulation. Means followed by the same letters within a column are not significantly different according to Duncan’s multiple range test (*p* < 0.05)

### Proteome profiles of flag leaves and grains under sink-source modifications

**Fig. 4:**
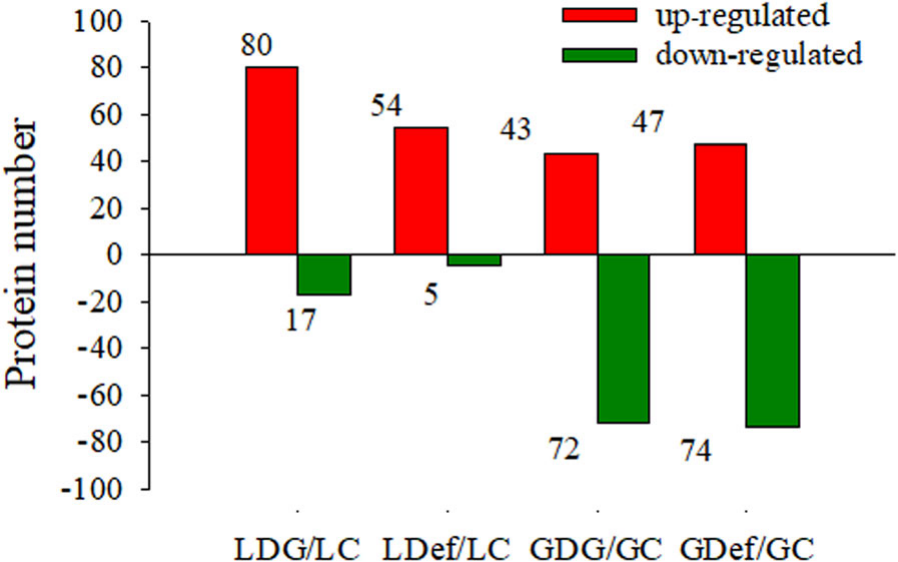
Number of differentially expressed proteins (DEPs) of the different groups. Data were obtained from two biological replicates. GC, grains in control plants; GDef, grains in defoliated plants; GDG, grains in de-grained plants; LC, leaves in control plants; LDef, leaves in defoliated plants; LDG, leaves in the de-grained plants

To investigate the proteome alterations of wheat flag leaves and grains due to different sink-source modifications, iTRAQ-based quantitative proteomics analysis was conducted. A total of 2821 proteins in flag leaves and 2467 proteins in grains were reliably identified at a global false-discovery rate (FDR) of 1%. In the LDG group, 97 proteins were assigned as differentially expressed compared with those in the intact control, including 80 increased and 17 decreased proteins. In the LDef group, 59 proteins were assigned as differentially expressed compared with those in the LC group, including 54 increased proteins and 5 decreased proteins. In the remaining grains of the half-degrained spike (GDG) group, 115 proteins were assigned as differentially expressed compared with those in the grain in control plants (GC) group, including 43 increased proteins and 72 decreased proteins. In the defoliated grain (GDef) group, 121 proteins were assigned as differentially expre**s**sed compared with those in the GC group, including 47 increased proteins and 74 decreased proteins (Fig. 4; Table S1).

### Gene ontology (GO) analysis

**Fig. 5:**
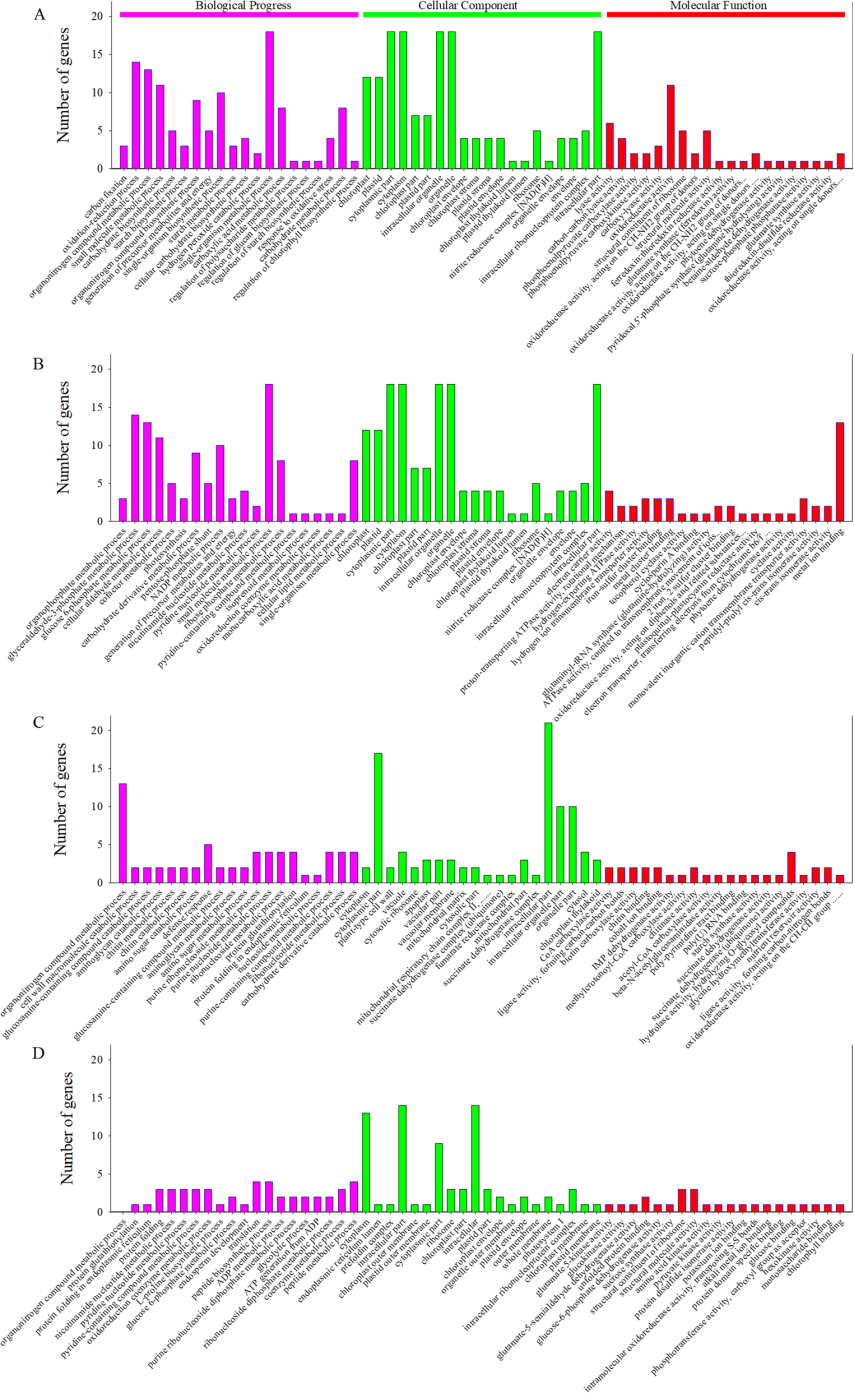
GO annotation of DEPs in manipulated flag leaf and grains compared to the control in biological processes, cellular components and molecular functions. (**a**), Flag leaf: half-degraining vs control; (**b**), flag leaf: defoliation vs control; (**c**), grain: half-degraining vs control; (**d**), grain: defoliation vs control

To illustrate the major responsive processes in flag leaves and grains, enrichment analysis was performed to assess the GO annotations. Differentially expressed proteins (DEPs) were clustered in the biological process (BP), cell component (CC), and molecular function (MF) categories according to GO analysis, and the top 20 GO terms are shown in Fig. 5.

BP analysis revealed that the DEPs identified in the comparison of the LDG with LC groups were mainly classified into carbon fixation, oxidation-reduction process, organonitrogen compound metabolic process, carbohydrate biosynthetic process, starch biosynthetic process, etc. According to MF analysis, the DEPs were classified into carbon-carbon lyase activity, oxidoreductase activity, structural constituent of ribosome, ferredoxin:thioredoxin reductase activity, ferredoxin-dependent glutamate synthase activity, glutamine hydrolysing activity, betaine-aldehyde dehydrogenase activity, etc. According to CC analysis, half-degraining mainly affected the chloroplast, plastid, cytoplasmic part, cytoplasm and intracellular organelles (Fig. 5a; Table S2).

In comparison of the LDef and LC groups, BP analysis revealed that the DEPs identified were mainly classified into organophosphate metabolic process, glyceraldehyde-3-phosphate metabolic process, glucose 6-phosphate metabolic process, photosynthesis, carbohydrate derivative metabolic process, etc. According to MF analysis, responses to defoliation mainly included the activities of electron carrier, proton-transporting ATPase, hydrogen-exporting ATPase, hydrogen ion transmembrane transporter, iron-sulfur cluster binding, tocopherol cyclase and glutaminyl-tRNA synthase (glutamine-hydrolyzing). Defoliation mainly affected chloroplasts, plastids, thylakoid membranes, chloroplast envelopes, organelle subcompartments and photosystem II (Fig. 5b; Table S2).

In comparison of the GDG and GC groups, BP analysis revealed that the proteins identified were mainly classified into organonitrogen compound metabolic process, cell wall macromolecule catabolic, amino sugar catabolic, defense response, aminoglycan metabolic, amino sugar metabolic, protein folding in endoplasmic reticulum (ER) and nucleoside metabolic processes. In comparison of the GDef/GC groups, the majority of identified proteins were classified into the activities of CoA carboxylase, ligase, forming carbon-carbon bonds, biotin carboxylase, methylcrotonoyl-CoA carboxylase, acetyl-CoA carboxylase, starch synthase, etc. According to CC analysis, half-degraining mainly affected the cytoplasm, cytoplasmic parts, vacuolar membrane, the mitochondria, intracellular parts and chloroplast thylakoids (Fig. 5c; Table S2).

According to BP analysis, grain responses to defoliation mainly included processes of organonitrogen compound metabolism, protein glutathionylation, protein folding in the ER, oxidoreduction coenzyme metabolism, L-proline biosynthesis, glucose 6-phosphate metabolism, endosperm development and others. According to MF analysis, responses to defoliation mainly included the activities of glutamate 5-kinase, glucokinase, glutamate-5-semialdehyde dehydrogenase, unfolded protein binding, glucose-6-phosphate dehydrogenase, sucrose synthase, structural constituent of ribosome, amino acid kinase, pyruvate kinase, protein domain specific binding and chlorophyll binding. Defoliation mainly affected the cytoplasm, ER lumen, cytoplasmic parts, ribosome, chloroplast parts and intracellular ribonucleoprotein complex (Fig. 5d; Table S2).

### Kyoto encyclopedia of genes and genomes (KEGG) pathway analysis

**Fig. 6:**
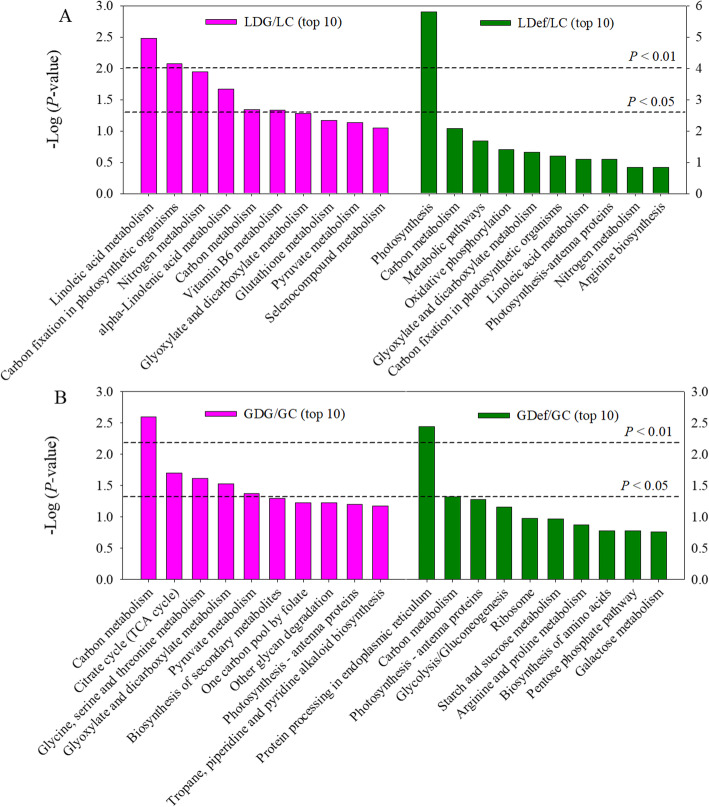
Most significant associated KEGG pathways of DEPs for LDG/LC (flag leaves of half-degrained group compared with control) and LDef/LC (flag leaves of defoliated group compared with control) (**a**) and GDG/GC (grains of half-degrained group compared with control) and GDef/GC (grains defoliated group compared with control) (**b**)

To understand the major responses of cellular processes to sink-source modifications in flag leaves and grains, the KEGG pathway database was used based on the large-scale molecular dataset. According to pathway enrichment analysis, the DEPs in the LDG group were significantly enriched in linoleic acid metabolism and α-linolenic acid metabolism, carbon fixation in photosynthetic organisms, nitrogen metabolism, carbon metabolism and vitamin B_6_ metabolism. The DEPs in the LDef group were significantly enriched in photosynthesis, carbon metabolism, metabolic pathways, oxidative phosphorylation, glyoxylate and dicarboxylate and metabolism (Fig. 6a). In comparison of the GDG group to the GC group, the DEPs were significantly enriched in four pathways in the KEGG database: carbon metabolism was the most represented pathway, followed by the citrate cycle (TCA cycle); glycine, serine and threonine metabolism;and glyoxylate and dicarboxylate metabolism. Most proteins in these pathways were decreased. In comparison of the GDef/GC groups, the DEPs were only enriched in two pathways, including protein processing in the ER and carbon metabolism (Fig. 6b). Taken together, the enrichment analyses based on proteomics data suggested that photosynthesis, carbon metabolism, and nitrogen/amino acid metabolism were the processes most responsive to sink/source manipulation.

### Enzyme activities

**Fig. 7:**
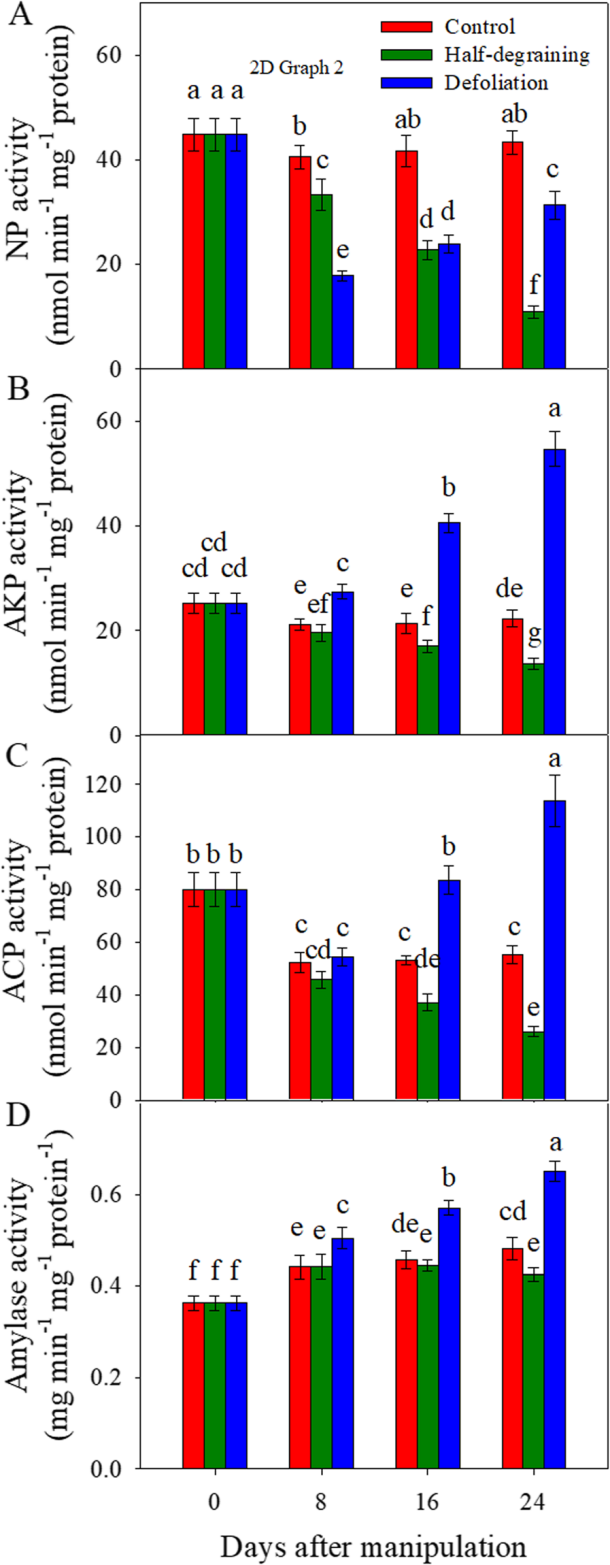
Comparison of the activities of neutral proteinase (**a**), alkaline proteinase (**b**), acid proteinase (**c**) and amylase (**d**) in flag leaves from the half-degraining, defoliation and control groups. Each value represents the mean ± SD from three independent samples. Columns labeled with different letters are significantly different at *p <* 0.05 according to Duncan’s test for multiple comparisons

During the experiment, the activities of neutral proteinase (NP), alkaline proteinase (AKP) and acid proteinase (ACP) (Fig. 7) in the flag leaves of intact plants remained relatively constant. Degraining decreased the NP (Fig. 7a), AKP (Fig. 7b) and ACP (Fig. 7c) activities at 8, 16 and 24 DAM compared with those in intact plants. Defoliation decreased NP activity at 8, 16 and 24 DAM but significantly increased AKP activity at 8, 16 and 24 DAM and ACP activity at 16 and 24 DAM.

Overall, the amylase activity in flag leaves was higher from 8 to 24 DAM than that at 0 DAM and remained constant from 8 to 24 DAM. From 8 to 24 DAM, defoliation significantly increased amylase activity compared with that of the control. No difference in amylase activity was observed at 8 and 16 DAM. At 24 DAM, the half-degraining treatment significantly decreased amylase activity (Fig. 7d).

### Grain mass responses to source and sink manipulations

The removal of spikelets on one side of spikes did not halve the grain number per spike (Table 3). Half-degraining increased the grain number per half-spike by 10.49% (*p* < 0.05) and the single grain mass by 8.54% (*p* < 0.05) compared with those of the control. As a consequence, half-degraining increased the grain mass per half-spike by 19.92%. Defoliation reduced the grain number per half-spike by 16.37% (*p* < 0.05). Grain mass also significantly responded to defoliation, which caused a reduction in grain mass per half-spike of 19.64% (*p* < 0.05). These data suggest that half-degraining reduces kernel abortion; hence, the number of grains per half spike after half-degraining was the highest of all treatments, and the inverse was true for after the defoliation treatment.

**Table 3:** Effects of half-degraining and defoliation on the grain number per half-spike, single grain mass and number of spikelets and grain mass per half-spike

	Grain number half-spike^−1^	Single grain mass (mg)	Grain mass half-spike^− 1^ (mg)
Control	17.35 b	44.27 b	768.08 b
Half-degraining	19.17 a	48.05 a	921.12 a
Defoliation	14.51 c	42.54 b	617.26 c

Data were obtained from three replicates. Means followed by the same letters within a column are not significantly different according to Duncan’s multiple range test (*p* < 0.05)

## Discussion

The source/sink interaction modulates leaf senescence, photosynthetic efficiency [20] and grain filling capacity, thus determining the yield of cereals [8, 21]. A strong source with sufficient reserves is necessary for grain filling, and a high sink capability promotes reserve remobilization from the source to the sink [22, 23]. In the present study, the values of NDVI, PRI and SPAD and chlorophyll fluorescence were determined from a single leaf, and we observed that half-degraining increased and defoliation decreased the levels of these indices compared to those in the intact control (Table 1; Fig. 1). Ultrastructural observations of flag leaf mesophyll cells at 16 DAM indicated that the state of chloroplasts retained better after half-degraining (Fig. 2b) but was inferior after defoliation (Fig. 2c). NDVI, PRI and SPAD are the most frequently used spectral indices and are associated with the chlorophyll content and leaf senescence in higher plants [24]. Chlorophyll fluorescence and chlorophyll degradation are also widely used as typical indicators of the progression of senescence and photosynthesis [19, 25–27]. Higher values of these indices are closely associated with grain yield [28]. Therefore, the evidence presented in the present study suggests that half-degraining may cause later leaf senescence, but defoliation may cause earlier leaf senescence.

It is well known that plant senescence is accelerated by ROS, while antioxidant enzymes play important roles in scavenging excess ROS and reduce detrimental effects of ROS on the cellular membrane [29, 30]. In wheat, the competence of the antioxidant defense system contributes to delayed senescence [31]. In the present study, the ROS content was higher in the LDef group than in the LC group, even though defoliation significantly increased the activities of SOD, POD and CAT, especially at 24 DAM, suggesting that increased levels of antioxidant enzymes may be a response to increased levels of ROS. In the DEPs identified using iTRAQ analysis, SOD (fragment) was upregulated in the LDef group at 16 DAM (Table S1). These results suggest that partial source removal may induce more active physiological responses and thus cause a burst of ROS and membrane oxidation, which in turn, result in premature senility of wheat plants. The increases in these antioxidant enzyme activities may be a strategy to protect plants from more serious damage, to partially relieve early senescence and to attempt to increase the active grain-filling period and grain mass. In contrast, the activities of SOD, POD and CAT decreased in the LDG group, but the ROS content also slightly decreased (Fig. 3; Table S1 for POD), indicating that half-degraining may impose a lower “burden” on flag leaves, resulting in lower ROS production.

Plant hormones and associated hormonal crosstalk are significantly related to modifications of the source/sink relationship, sink capacity, grain-filling rate [32–34], membrane stability, foliar senescence and photosynthetic efficiency, possibly by acting as signals to scavenge ROS or prevent ROS formation [35–37].

Cytokinins are the most significant phytohormones that regulate the initiation and timing of leaf senescence [10, 38, 39] and are closely associated with the progress of leaf senescence in the stay-green mutant *tasg1* of wheat [31] and in the stay-green phenotype of maize (*Zea mays*) [40]. The grain CTK contents are positively associated with the maximum grain mass [35, 41, 42]. In main cereal crops including wheat, a higher CTK concentration is found in the endosperm of grains and functions during cell division there at the early stages of grain development, which, in combination with the functions of auxin, are positively and significantly correlated with the rate of both cell division and grain filling [11, 34, 39, 42–45] due to the enhanced sink strength [46, 47].

The grain IAA concentration rapidly increases in maize at early grain filling [48], thus creating an “attractive power” and leading to an increase in the CTK concentration in grains [49, 50], which is associated with an increase in assimilate transport to developing grains [48]. In addition, auxin is linked with an enhanced sink capacity, resulting in increases in nutrient assimilation source nutrient remobilization, cell enlargement and the grain-filling rate [51, 52].

In our study, defoliation decreased the leaf/grain ratio CTK and IAA concentrations (Table 2). Based on results from current and previous studies, we postulate that higher source competition due to defoliation relatively stimulates hormone biosynthesis in grains, thereby increasing the sink strength and enhancing their potential to absorb more reserves from the limited source. Meanwhile, relatively lower levels of CTKs and IAA in defoliated flag leaves promote the degradation of large molecules for grain filling, which causes early senescence, as demonstrated by the ultrastructural observations and measurements of the vegetation indices (Table 1; Fig. 2). This view is highly consistent with the findings that indicate that in cultivars with larger sink ratios, there is greater competition for the source photosynthate supply to meet grain filling, leading to premature senescence [21]. Furthermore, the data presented in the present study suggest that the leaf /grain ratio of the CTK and IAA concentration may play a dominant role in regulating leaf senescence in wheat.

Half-degraining increased the leaf/grain ratio of CTK concentration (Table 2). Based on these results and as discussed above, we suggest that the relatively high source strength due to half-degraining and thus the lower competitive pressure for source reserves cause a higher leaf/grain ratio of CTKs and a slower remobilization of leaf reserves, which contribute to delayed leaf senescence.

CTKs and JA are the major hormones that have antagonistic signaling effects on plant senescence [13]. The accumulation of ROS and JA leads to early leaf senescence and a low photosynthesis efficiency [53]. Specific inhibition of JA biosynthesis in the leaf results in an increase in the grain yield of rice [54]. In our study, we observed that half-degraining decreased the leaf/grain ratio of JA concentration at 8 DAM and defoliation decreased this ratio during the experimental period (Table 2). Considering the response of JA to source manipulation, we hypothesize that the lower leaf/grain ratio of JA may have contributed to avoid premature leaf senescence in defoliated plants.

Leaf senescence is associated with fundamental changes in the proteome. In the present study, comparative proteome analysis based on iTRAQ was performed to identify the critical candidate factors involved in leaf senescence and grain filling. The GO terms within the BP, MF and CC categories are shown in Fig. 5. KEGG pathway enrichment analysis of these genes was also performed (Fig. 6).

The chloroplast is the central organelle that produces ROS, whereas accumulation of ROS may cause oxidative damage and inhibit photosynthesis [55]. Ferredoxin:thioredoxin reductase is required for chloroplast development in *Arabidopsis thaliana* [56] and acts as a component of antioxidative defense systems [57]. The destruction of the oxidation-reduction system causes a burst of ROS, such as superoxide (O_2_^·−^) and hydrogen peroxide (H_2_O_2_), leading to early leaf senescence [58, 59]. Glutathione is recycled through the oxidation/reduction process and is thus involved in oxidative stress; its metabolism plays an important role in maintaining the redox balance, eliminating oxidative damage [60]. Phytoene dehydrogenase is associated with the biosynthetic pathway of carotenoid, which serves as a membrane antioxidant [61]. In comparison between the LDG and LC groups, ferredoxin:thioredoxin reductase was clustered in MF and was upregulated in the LDG group. The most prevalent GO terms for BP were involved in ROS metabolic processes, including oxidation-reduction process, response to oxidative stress, H_2_O_2_ metabolic process and O_2_^·−^ removal (Table S1). In these processes, the homeostasis of the oxidation-reduction process was most strongly enhanced in the LDG group, as judged by the PAS_Zscore (Table S2). According to MF analysis, the activities of phytoene dehydrogenase and several kinds of oxidoreductases were significantly affected, and glutathione metabolism was identified in the top 10 KEGG pathways (Fig. 5a). Thylakoid-bound ascorbate peroxidase (APX) (fragment) and betaine aldehyde dehydrogenase (BADH; an important enzyme for betaine biosynthesis) were upregulated (Table S1) and involved in many pathways (Fig. S1). Both APX and betaine function as ROS scavengers [62]. These results imply that half-degraining may enhance the antioxidant ability of leaves by promoting ROS scavenging activities and may, at least, partially contribute to the delayed leaf senescence (Table 1; Fig. 2).

KEGG pathway analysis of the LDG/LC groups suggested that the DEPs were mainly involved in nitrogen and carbon metabolism. Correspondingly, MFs, including the glutamate synthase, ferredoxin-dependent glutamate synthase (Fd-GOGAT; EC 1.4. 7.1) and pyridoxal 5′-phosphate synthase (glutamine hydrolyzing) activities and carbon metabolism (Fig. 5a; Table S2), were affected. BP analysis showed that these processes were upregulated, indicating that nitrogen and carbon metabolism may be enhanced in the LDG group. Indeed, according to CC analysis, the categories that play important roles in protein biosynthesis, such as the cytosolic ribosome and ribosome, were highly represented in the LDG group (Fig. 5a; Table S2). Accordingly, DEPs such as 60S ribosomal and 40 S ribosomal proteins were upregulated (Table S1).

In comparison between the LDG and LC groups, the DEPs related to photosynthesis and generation of precursor metabolites and energy were upregulated (Table S1) and showed a significant interaction with carbon fixation in photosynthetic organisms (Fig. S1B). MFs, such as the activities of phosphoenolpyruvate carboxykinase, and sucrose-phosphate phosphatase, were affected (Table S1). Most importantly, sucrose phosphate phosphatase is associated with source activity, enhances the transport of its resulting product sucrose from leaf into the sink [63] and affects the progress of leaf senescence in wheat [64].

In the GDG group, amine oxidase, which catalyzes the reaction to produce H_2_O_2_ [65], was downregulated compared with that in the GC group, while Cu-Zn SOD and dehydroascorbate reductase (DHAR) were upregulated (Table S1). SOD is an important antioxidant enzyme, and DHAR reduces the level of leaf ROS through its recycling of ascorbic acid, a major antioxidant in plants and as a consequence, influences the rate of leaf senescence and photosynthetic activity [29, 30]. Accordingly, GO analysis showed that the primary amine oxidase and oxidoreductase activities were affected. Protein glutathionylation, a mechanism for redox regulation and signaling [66], was upregulated (Table S2). These changes suggest that the remaining grains may have a higher ability to scavenge ROS in half-degrained plants with higher source/sink ratio.

Protein disulfide isomerases (PDIs) are enzymes found primarily in the ER in eukaryotes and play a vital role in protein folding. The levels of PDIs are associated with the activity of protein biosynthesis [67–69] and abiotic stress resistance [68]. In the present study, the levels of PDIs were upregulated in the GDG group (Table S1), and the 60S ribosomal protein L29, 40S ribosomal protein S12 and eukaryotic translation initiation factor 4B1 were also upregulated (Table S1). These changes indicate de-graining may affect protein synthesis in the grains of GDG group.

Photosynthetic processes, including photosynthesis and the light reaction, light harvesting and the dark reaction, were also significantly upregulated; in addition, plastocyanin and *rbcL* gene products (fragments) were upregulated in the GDG group, indicating a possible higher use efficiency of light. The *rbcL* gene is involved in carbon, glyoxylate and dicarboxylate metabolism [70]. These changes indicate that half-degraining affect metabolism of carbon, glyoxylate and dicarboxylate through the expression of *rbcL* gene as shown in Fig. S1C.

In plants, acetyl-CoA carboxylase catalyzes the first committed step of de novo fatty acid biosynthesis via the carboxylation of acetyl-CoA to malonyl-CoA [69, 71, 72]. In the present study, acetyl-CoA carboxylase, plastid acetyl-CoA carboxylase (fragment) and acetyl-coenzyme A carboxylase (fragment) were downregulated in the GDG group; the activities of CoA carboxylase, acetyl-CoA carboxylase and biotin carboxylase were also affected. Plastid acetyl-CoA carboxylase showed interactions with many other DEPs (Fig. S1C). These results indicate that fatty acid biosynthesis may be affected in the GDG group.

In the analysis of the MF category for the GDG/GC group, processes involved in succinate dehydrogenase (ubiquinone) activity were identified (Fig. 5c; Table S2), in accordance with the upregulation of the succinate dehydrogenase [ubiquinone] flavoprotein subunit and mitochondria and as represented by MF categories such as mitochondrial electron transport, succinate to ubiquinone (Fig. 5c) and citrate cycle (Fig. 6b). These processes might occur in CCs, such as the mitochondrial matrix, mitochondrial respiratory chain complex II, and succinate dehydrogenase complex (ubiquinone) (Fig. 5c). In addition, the GMP biosynthetic process, GMP metabolic process, hydrolase activity (hydrolyzing O-glycosyl compounds), glycosyl compound metabolic process and glycosyl compound biosynthetic process were significantly represented (Table S2). These results indicate that half-degraining may affect energy generation in the remaining grains. In the BP analysis, we indeed observed that categories related energy generation were upregulated, including generation of precursor metabolites and energy, and electron transport coupled ATP synthesis coupled electron transport (Table S2).

In our study, the relative ROS content increased in the LDef group (Fig. 3), which might inhibit photosynthesis [56], as discussed above. However, glutathione s-transferase, superoxide dismutase (fragment), and oxidoreductase activity, which act on superoxide radicals as acceptors; SOD activity (Fig. 3); oxidation-reduction process; oxidoreduction coenzyme metabolic process; and tocopherol cyclase and glutathione S-transferase (Table S1) and thus tocopherol cyclase activity were upregulated (Table S2), indicating that in the LDef group, some strategies may have developed to help plants scavenge ROS and eliminate oxidative damage [73, 74]. However, the overall capability of ROS metabolic processes, removal of superoxide radicals, cellular response to superoxide, cellular response to oxygen radicals, regulation of H_2_O_2_ metabolic process and cellular response to ROS were downregulated (Table S2).

Interestingly, the top 20 significantly represented BPs were upregulated in the LDef group (Table S2). GO terms related to photosynthesis were significantly enriched but showed different changing trends. For instance, plastid organization and cofactor metabolic processes were upregulated. DEPs related to photosynthesis-antenna proteins and carbon metabolism were also upregulated (Fig. S1B). However, chlorophyll biosynthetic, chlorophyll metabolic and pigment biosynthetic processes were downregulated (Table S2). These results indicate that leaf photosynthesis may be affected (Fig. 5b). According to MF analysis, iron-sulfur cluster binding, 2Fe-2S iron-sulfur cluster binding and 3Fe-4S iron-sulfur cluster binding, which play important roles in the photosynthetic rate by regulating electron transfer and the chlorophyll content in rice [75], were significantly affected. Accordingly, many MF categories, including the activities of electron carrier, proton-transporting ATPase, hydrogen-exporting ATPase, hydrogen ion transmembrane transporter and plastoquinol-plastocyanin reductase, were changed (Fig. 5b; Table S2), indicating that the energy supply might be very important in the LDef group. Indeed, the GO categories that were enriched and primarily related to energy metabolism were upregulated, including the glyceraldehyde-3-phosphate metabolic process, glucose 6-phosphate metabolic process, carbohydrate derivative metabolic process and generation of precursor metabolites and energy via the pentose-phosphate pathway and pentose-phosphate shunt (Table S2).

In the LDef group, peptidyl-prolyl *cis*-*trans* isomerase and peptidylprolyl isomerase were enriched, which could affect protein folding and interact with the chlorophyll a-b binding protein and cytochrome *b-c1* complex subunit Rieske as shown in Fig. S1B. Regarding lipid metabolism, organophosphate metabolic, cellular lipid metabolic, lipid biosynthetic and phospholipid biosynthetic processes were also upregulated in the LDef group. In addition, protein glutathionylation, a mechanism of redox regulation and signaling [66], and oxidation-reduction processes were downregulated, indicating a possible decrease in the ability to scavenge ROS.

In BP category analysis of the GDef group, organonitrogen compound metabolic and cellular protein metabolic processes were enhanced. However, protein folding was downregulated (Table S2); in addition, translation, peptide biosynthetic, peptide metabolic and L-proline biosynthetic processes were downregulated. As a consequence, a large number of low molecular weight glutenins (subunits) were decreased (Table S2). These processes may occur in the ER lumen, prefolding complex and ribosome, regulating the structural constituents of ribosomes (Table S2), especially in the ER, because protein processing in the ER was significantly affected (Fig. 6b).

In the GDef group, the glucose 6-phosphate metabolic process and the generation of precursor metabolites and energy were downregulated (Table S2). Pyruvate kinase was decreased, and pyruvate kinase activity was decreased (Table S1, S2). At the same time, the chlorophyll a-b binding protein (chloroplastic) was also downregulated. Furthermore, photosynthesis, light harvesting and the light reaction were also decreased. These results indicate that in the grains of defoliated plants, the photosynthetic efficiency and energy generation may be affected.

Based on these results and those discussed above, we further surmise that defoliation hinders endosperm development, seed development and maturation, as shown in Table S2.

A large amount of macromolecules in normal senescent leaves are degraded into small molecules by degradation enzymes [76], which leads to a massive remobilization of phloem-mobile nutrients from senescing plant parts to developing sinks [22, 77].

As suggested by the proteomic analysis results, the source-sink modifications mainly affected nitrogen and carbon metabolism and energy production. During leaf senescence in plants, genes encoding proteases are overexpressed [78–81]. A large number of senescence-associated proteases are upregulated, and the resulting catabolic products are mobilized from leaves to developing grains [76, 82–84]. The activity of amylases plays a vital role in starch degradation and the remobilization of nonstructural carbohydrates from vegetative organs to developing sinks and is thus essential for grain filling [85, 86]. In the present study, defoliation increased the activities of amylase (Fig. 7) and acid and alkaline proteinases (Fig. 7). Half-degraining decreased proteinase activity at 16 and 24 DAM and amylase activity at 24 DAM. These results indicate that the earlier senescence of defoliated plants might be partially due to the increases of the activities of these hydrolytic enzymes and the earlier nutrient remobilization from a smaller leaf area in defoliated plants, while the delayed senescence in half-degrained plants might be partially caused by decreased protein and starch degradation.

Vegetative organ CTKs play an important role in regulating senescence, which is associated with a delay in proteolytic activity and thus nitrogen remobilization to developing grains [87–89]. Therefore, we speculate that the decrease in the CTK concentration may be associated with the increases in proteolytic activity in the LDef group.

## Conclusions

In this study, the reduced sink/source ratio due to half-degraining delayed wheat leaf senescence, while the higher sink/source ratio due to defoliation caused a higher amount of ROS production and facilitated the degradation of chlorophyll-protein complexes and carbohydrates and thus plant senescence. CTKs, IAA and JA and the interactions among these hormones played major roles in regulating the source capability and sink strength, thereby impacting the process of leaf senescence. Sink and source manipulations induced many differentially expressed proteins, which were mainly involved in ROS scavenging, leaf photosynthesis, carbon and nitrogen metabolism, generation of precursor metabolites and energy and grain development. Degradation of carbohydrates and proteins by hydrolytic enzyme activities was promoted in the flag leaves of defoliated plants but retarded in those of half-degrained plants, which in turn, impacted carbon and nitrogen metabolism in grains. Half-spike removal enhanced but defoliation depressed single grain growth, indicating that the yield potential of wheat is limited by both the sink capacity and source availability. Our results indicate that future yield improvements may be able to be achieved by strengthening both the source and sink capacities during breeding to increase the yield potential in wheat.

## Methods

### Site, experiment and design

The experiments were conducted in the 2017–2018 cropping season in a field at an experimental station (36°42′N, 117°4′E; altitude 48 m) of the Shandong Academy of Agricultural Sciences, China. The soil was a fine loam. Wheat cultivar Jimai 23, which was developed by the Crop Research Institute, Shandong Academy of Agricultural Sciences, was used in this experiment. Jimai 23 is a representative of varieties that are popularly grown in northern China due to their high grain yield. Seeds were sown on Oct. 10, 2017, at a rate of 225 seeds m^− 2^ in three blots (each of 30 m^2^). Plots were fertilized before planting with 10 g m^− 2^ P_2_O_5_, 10 g K_2_O m^− 2^ and 7.5 g N m^− 2^. At the shooting stage (Zadoks stage 31), 15 g N m^− 2^ was top-dressed with urea. Matured grains were harvested on Jun. 6, 2018.

### Source and sink manipulations

Sink-source manipulations were conducted as follows: (1) all spikelets were manually removed from one side of the spikes to double the assimilate availability for the remaining grains; (2) all leaves of culms except flag leaves were removed to reduce the source assimilate availability. Each manipulation was performed in three 2-m sections of the rows at 2 days after anthesis in each plot. Three 2-m sections were selected and were left intact as a control.

### Measurement of vegetation index

NDVI and PRI were measured with a PlantPen instrument (Photon Systems Instruments, Brno, Czech Republic) from 30 flag leaves per plot. The SPAD index was determined from at least 30 flag leaves per plot using a chlorophyll meter (SPAD-502 plus, Konica Minolta, INC., Japan) at 8, 16 and 24 DAM. Data from each plot were averaged to obtain themean.

### Chlorophyll fluorescence assay and imaging

Chlorophyll fluorescence analysis was performed at different stages to determine *Fv*/*Fm* and Φ_PSII_ in flag leaves using a kinetic imaging fluorometer (FluorCam, Photon System Instruments Ltd., Brno, Czech Republic) as described by Kong et al. [90].

### Transmission Electron microscopy (TEM)

TEM observations were processed as reported previously by Kong et al. [90] using a transmission electron microscope (JEM-1200EX; JEOL Ltd., Tokyo, Japan) at 80 kV.

### Hormone analysis

Flag leaves or spikes in triplicate from each treatment were collected from three biological replicates at 8, 16 and 24 DAM; immediately frozen in liquid nitrogen; and then stored at − 80 °C. Then, the leaves and grains were used for each hormone analysis.

The extraction and purification of zeatin, zeatin riboside, kinetin, and IAA were performed according to Liu et al. [34]. Briefly, samples (approximately 0.10 g flag leaves or grains) were ground in a pre-cooled mortar containing 5 ml of an 80% (v/v) methanol extraction solution and 1 mM butylated hydroxytoluene was added as an antioxidant. The resulting extracts were incubated overnight at 4 °C and then centrifuged at 12000×*g* for 15 min at 4 °C. The supernatants were dried with N_2_ at 40 °C, dissolved in 200 μl methanol and filtered through a membrane (0.45 μm). High-performance liquid chromatography (HPLC; Rigol L3000, Beijing, China) with a Kromasil C18 reversed-phase column was used to measure the hormone concentration. The mobile phase was prepared by mixing methanol and ultrapure water at a ratio of 2:3 (v/v). The injection volume was 10 μl, the flow rate was 0.8 ml/min, the column temperature was 35 °C, the elution time was 60 min, and the detection wavelength was 254 nm. Three independent biological replicates of each sample were performed.

For JA analysis, the sample (approximately 0.10 g) was ground to a fine powder in liquid nitrogen and extracted with 1.0 ml of 90% (v/v) methanol overnight at 4 °C. The crude extract was centrifugated at 12000×*g* for 10 min and the supernatant was collected. The resulting precipitate was then re-extracted with 0.5 ml of 90% methanol for 2 h and recentrifuged. After two times of extractions, the supernatants were combined and air-dried by incubation at 40 °C. The dried samples were dissolved in 1 ml of ethylacetate/cyclopentane (1:1, v:v) and 20 μl of 1 mg/ml trichloroacetic acid, followed by vortexing for 30 min and centrifuged at 8000×*g* for 10 min. The top organic phase was dried, dissolved in mobile phase and injected into a reverse phase C18 HPLC column equipped with a fluorescence detector for analysis of JA. The mobile phase was prepared by mixing methanol and 0.1% formic acid (65%:35%, v/v). The injection volume was 10 μl, flow rate was 0.8 ml/min, column temperature was 35 °C, and elution time was 30 min; 230 nm was monitored. At least three independent biological replicates of each sample were performed.

### Protein preparation

Leaves (approximately 0.1 g FW for each biological replicate) were ground into a fine powder in liquid nitrogen and thoroughly transferred to an Eppendorf tube. To the tube, 1 ml of precooled phenol extraction buffer (100 mM Tris-HCl, 50 mM EDTA, 100 mM KCl, 2% (w/v) DTT, 30% sucrose and 2% SDS; pH 8.0) was added, and the mixture was incubated at room temperature for 10 min. Then, 1 ml phenol saturated with Tris-HCl (pH 8.0) was added. The mixture was shaken for 40 min at 4 °C. After centrifugation at 12000×*g* for 15 min at 4 °C, the upper phenolic phase was collected, debris was removed, and protein was precipitated with precooled 100 mM ammonium acetate-methanol solution for 12 h at − 20 °C. After centrifugation, the pellet was washed three times with cold acetone and air-dried for 5 min. Protein was resuspended in 600 μl of lysis buffer (4% SDS, 100 mM Tris-HCl, 1 mM DTT, pH 7.6), incubated for 60 min at room temperature and centrifuged at 12000×*g* for 10 min at room temperature. The sample was collected and stored at − 80 °C for iTRAQ analysis. The protein concentration was determined according to the Bradford assay (Bradford, 1976).

### Trypsin digestion and iTRAQ labeling

Approximately 100 μg of protein from each biological replicate was used for digestion. First, the protein sample was reduced by the addition of 120 μl buffer (10 mM DTT, 8 M urea, 100 mM tetraethylammonium bromide (TEAB), pH 8.0) at 60 °C for 1 h and then alkylated using 50 mM iodoacetamide for 40 min at room temperature in the dark. Subsequently, the proteins (precisely 0.1 mg) were diluted with 100 μl 100 mM TEAB. Then, 2 μl sequencing-grade trypsin (1 μg/μl; Promega) was added for digestion at 37 °C for 12 h. After centrifugation at 12000×*g* for 20 min, the supernatant was collected and lyophilized. The lyophilized sample was thawed and reconstituted in 100 μl 100 mM TEAB. The 100 μl iTRAQ reagent was transferred to the sample tube and labeled by incubation for 2 h at room temperature. After the addition of 200 μl water to quench the labeling reaction, the solution was lyophilized.

### Mass spectrometry (MS) analysis

The labeled peptides were subjected to a nanospray Flex source and analyzed by a Q-Exactive mass spectrometer (Thermo, USA). The following analyses were performed according to the procedures as described by Xu et al. [91] and Xiong et al. [92].

### Protein identification and quantification

The resulting MS/MS spectral data files were analyzed using Proteome Discoverer™ 1.3 (Thermo, USA) software using the SEQUEST® search engine and were searched against the UniProt *Triticum aestivu*. FASTA database for wheat at a 1% FDR. The mass errors of precursor ions and fragment ions were set to 10 ppm and 0.02 Da, respectively.

The screening criteria for reliable proteins were as follows: unique peptide ≥1, removal of invalid values and antilibrary data, and screening of differentially expressed proteins based on reliable proteins. To screen differential proteins, Student’s *t*-test *p* < 0.05 and fold changes > 1.3 or < 0.77 across the flag leaf samples or more than 1.5-fold or less than 0.67-fold were selected across the grain samples from a control, and two treatments were applied based on two experiments.

### Bioinformatics analysis

A database (http://www.omicsbean.com:88/) and the OmicsBean software (http://www.ebi.ac.uk/interpro/) were used for gene ontology (GO) annotation. In the OmicsBean software, each protein was assigned to biological processes, cellular components and molecular functions. After annotation, proteins were mapped to pathways in the Kyoto Encyclopedia of Genes and Genomes (KEGG) database (http://www.kegg.jp/). PPI analysis was also performed using Cytoscape software.

### Enzyme assays

For total amylase activity, flag leaves (approximately 0.1 g FW) were collected from three replicates and homogenized in a prechilled mortar and pestle with 1 ml cooled distilled water. After adding another 9 ml of distilled water, the mixture was placed at room temperature for 20 min to extract total amylase. Then, the homogenate was centrifuged at 12000×*g* for 15 min at 4 °C. The supernatant was separated and used as the enzyme extract. To 1 ml enzyme extract, 1 ml soluble starch (1%, w/v) or 1 ml distilled water (control) was added and incubated at 40 °C for 5 min. Subsequently, 2 ml of 3,5-dinitrosalicylic acid reagent (1% (w/v) 3,5-dinitrosalicylic acid and 100 mM phosphate buffer (pH 7.0)) was added to the mixture before heating in a boiling water bath. Absorbance was measured at 540 nm using a spectrophotometer. The enzyme activity is expressed as the amount of enzyme catalyzing the production of 1 mg reducing sugar per min per mg protein.

Protease (EC 3.4.21.40) activity was spectrophotometrically determined using casein as the substrate. Flag leaves (approximately 0.1 g FW) were ground in a mortar. The homogenates were centrifuged at 12000×*g* for 15 min at 4 °C, and the supernatant was transferred to a test tube for the measurement of protease activity. The reaction mixture containing 1 ml enzyme extract and 1 ml casein (2%) was heated at 40 °C for 10 min. A trichloroacetic acid solution (2 ml 5%) was added to the reaction for 20 min at 40 °C. After centrifugation (12,000×*g*, 10 min), a reaction mixture containing 1 ml supernatant, 5 ml Na_2_CO_3_ (0.5 M), and 1 ml Folin reagent was prepared, and its absorbance was then colorimetrically measured at 680 nm. The pH of the incubation media for acid proteinase, neutral proteinase, and alkaline proteinase buffer solutions were 3.6, 7.5 and 11, respectively. The activities of proteases were estimated by measuring tyrosine and other aromatic amino acids released from hydrolyzed proteins according to a standard curve. Activity is expressed as nmol tyrosine released per min per mg protein.

### Grain mass

Eighty culms were harvested from each plot at maturity and threshed by hand. The grains were oven-dried at 60 °C to a constant weight. Grains were then weighed, and the grain number was counted to obtain single grain mass. The grain mass and grain number were determined based on half-spikes.

### Statistical analysis

For the physiological indices obtained, statistical analyses were performed using the software of the data processing system (DPS) (v.14.10, Refine Information Tech. Co., Ltd., Hangzhou, Zhejiang, China). Duncan’s multiple range test was used to evaluate the statistical significance of the results.

## Supplementary information

**Additional file 1 Table S1.** Detailed information of the DEPs identified by iTRAQ in the LDG and LDef groups in comparison with the LC group and in the GDG and GDef groups in comparison with the GC group. GC, grains in control plants; GDef, grains in defoliated plants; GDG, grains in de-grained plants; LC, leaves in control plants; LDef, leaves in defoliated plants; LDG, leaves in the de-grained plants

**Additional file 2 Table S2.** Two manipulation-related DEPs identified in GO categories and were grouped into three levels: biological process (BP), cellular component (CC) and molecular function (MF)

**Additional file 3 Fig. S1.** Interaction networks of the differentially expressed proteins in comparison of LDG/LC (A), LDef/LC (B), GDG/GC (C) and GDef/GC (D). GC, grains in control plants; GDef, grains in defoliated plants; GDG, grains in de-grained plants; LC, leaves in control plants; LDef, leaves in defoliated plants; LDG, leaves in the de-grained plants. Circle nodes denote differentially expressed proteins (genes), and colored rectangles indicate KEGG pathways. PPI analysis was performed using Cytoscape software, in which the threshold value (confidence cutoff) was set at 400, when the confidence score of the potential PPI was high, as indicated by solid lines or dashed lines. A solid line between two proteins indicates a known interaction annotated in the database; a dashed line between proteins indicates a potential interaction

## Data Availability

The datasets generated and analyzed during the present study and the plant materials used in the present study are available from the corresponding author upon reasonable request.

## Abbreviations

ACP: Acid proteinase
AGC: Automatic gain control
AKP: Alkaline proteinase
BADH: Betaine aldehyde dehydrogenase
BP: Biological process
CAT: Catalase
CC: Cell component
CTKs: Cytokinins
DAM: Days after manipulation
DEP: Differentially expressed protein
DHAR: Dehydroascorbate reductase
DPS: Data processing system
DTT: Dithiothreitol
EDTA: Ethylene diamine tetraacetic acid
Fd-GOGAT: Ferredoxin-dependent glutamate synthase
FDR: False-discovery rate
ER: Endoplasmic reticulum
*Fv*/*Fm*: Maximum PSII quantum yield
GC: Grains in control plants
GDef: Grains in defoliated plants
GDG: Grains in de-grained plants
H_2_O_2_: Hydrogen peroxide
HPLC: High-performance liquid chromatography
IAA: Indoleacetic 3-acid
iTRAQ: Isobaric tag for relative and absolute quantitation
JA: Jasmonic acid
KEGG: Kyoto encyclopedia of genes and genomes
LC: Leaves in control plants
LDef: Leaves in defoliated plants
LDG: Leaves in de-grained plants
MF: Molecular function
MS: Mass spectrometry
NDVI: Normalized difference vegetation index
NP: Neutral proteinase
O_2_^·−^: Superoxide
PDI: Protein disulfide isomerase
POD: Peroxidase
PPI: Protein-protein interaction
PRI: Photochemical reflectance index
SDS: Sodium dodecyl sulfate
SOD: Superoxide dismutase
SPAD: Soil and plant analyzer development
TEAB: Tetraethylammonium bromide
Φ_PSII_: Effective PSII quantum yield
