# Supplementary figures and images for: Source-sink modifications affect leaf senescence and grain mass in wheat as revealed by proteomic analysis
Source: BMC Plant Biol. 2020 Jun 5;20:257. doi: 10.1186/s12870-020-02447-8 (PMC7275590; doi:10.1186/s12870-020-02447-8)

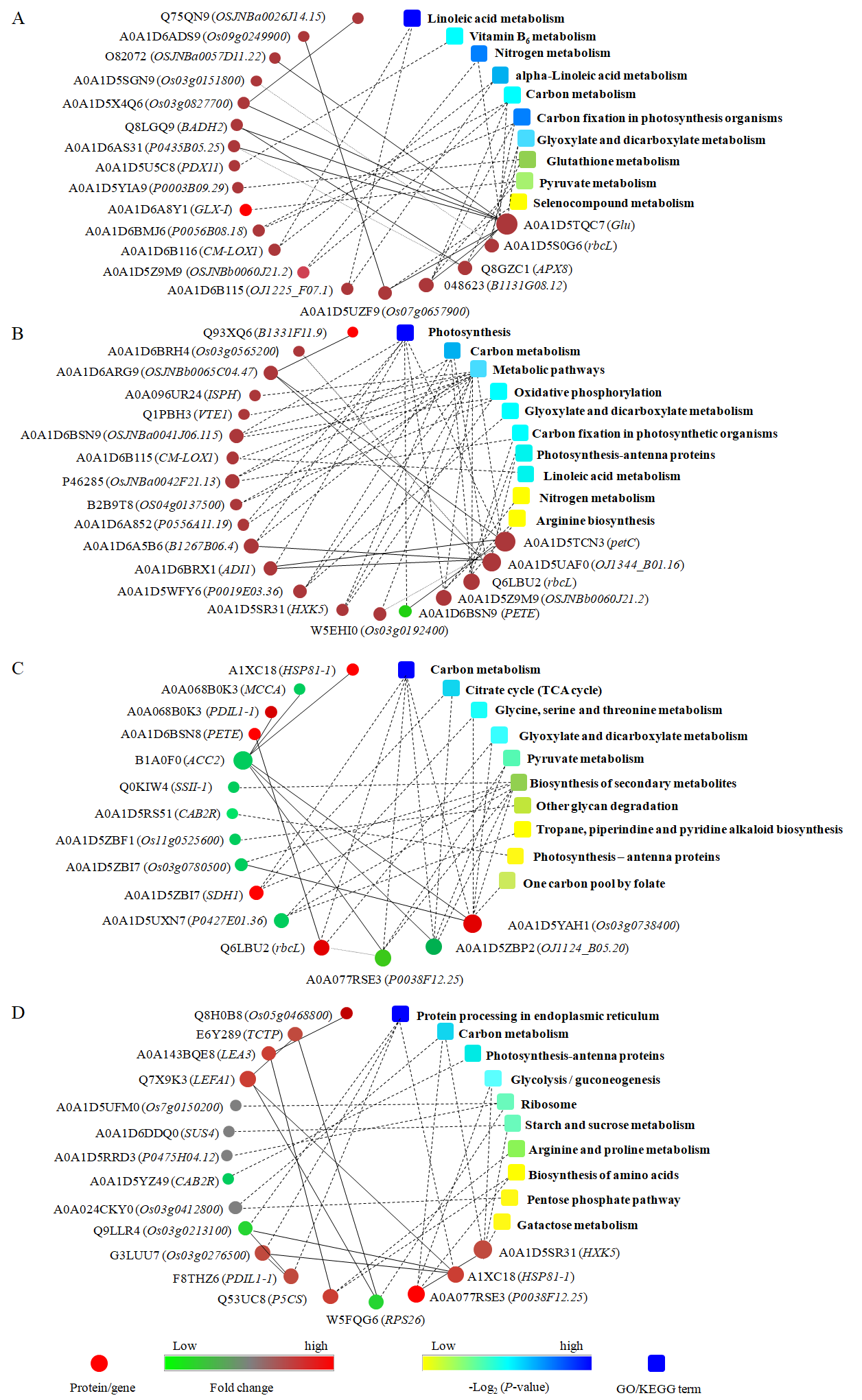

Supplement: Supplementary file 3 — Additional file 3 Fig. S1. Interaction networks of the differentially expressed proteins in comparison of LDG/LC (A), LDef/LC (B), GDG/GC (C) and GDef/GC (D). GC, grains in control plants; GDef, grains in defoliated plants; GDG, grains in de-grained plants; LC, leaves in control plants; LDef, leaves in defoliated plants; LDG, leaves in the de-grained plants. Circle nodes denote differentially expressed proteins (genes), and colored rectangles indicate KEGG pathways. PPI analysis was performed using Cytoscape software, in which the threshold value (confidence cutoff) was set at 400, when the confidence score of the potential PPI was high, as indicated by solid lines or dashed lines. A solid line between two proteins indicates a known interaction annotated in the database; a dashed line between proteins indicates a potential interaction [file 12870_2020_2447_MOESM3_ESM.tif]
